# Biomimetic nanoparticles in cancer photodynamic therapy: a review of targeted delivery systems and therapeutic outcomes

**DOI:** 10.3762/bjnano.17.27

**Published:** 2026-03-05

**Authors:** Valentina I Gorbacheva, Alexey S Grabovoy, Polina S Marukhina, Anastasiia O Syrocheva, Ekaterina P Kolesova

**Affiliations:** 1 Sirius University of Science and Technology, 1 Olympic Avenue, Sirius, Krasnodar Region, 354340, Russia

**Keywords:** biomimetic nanoparticles, immunogenic cell death, nanocarriers, photodynamic therapy, targeted drug delivery, tumor hypoxia modulation

## Abstract

Photodynamic therapy (PDT) is a minimally invasive cancer treatment that uses photosensitizers (PSs) activated by light to produce cytotoxic reactive oxygen species (ROS). Although PDT shows clinical promise, its effectiveness is limited by factors such as insufficient tumor targeting, tumor hypoxia, PS instability, and weak immune responses. Biomimetic nanoparticles (BNPs), which combine natural biological materials like cell membranes with synthetic nanocarriers, have emerged as versatile platforms to overcome these challenges. BNPs improve PDT by enhancing tumor-specific delivery of PSs, relieving hypoxia through oxygen delivery or catalytic oxygen generation, and boosting antitumor immunity by promoting immunogenic cell death and working synergistically with immune checkpoint inhibitors. This review details recent progress in BNP-based strategies for targeted PS delivery, ROS production enhancement, hypoxia modulation, and immune system activation. Additionally, it explores multifunctional and theranostic nanoplatforms, their applications in various cancers, and advances toward clinical use. By integrating targeted delivery, tumor microenvironment modulation, and immunotherapy, BNP-facilitated PDT holds great potential for advancing precise cancer treatments.

## Review

### Introduction

Cancer remains a formidable global health challenge, demanding innovative therapeutic strategies that balance efficacy with reduced systemic toxicity [1]. Among emerging treatments, photodynamic therapy (PDT) has garnered considerable attention due to its minimally invasive nature, spatiotemporal control, and ability to selectively destroy tumor tissues while sparing healthy cells [2]. PDT operates through a unique mechanism involving three key components: a photosensitizer (PS), light of a specific wavelength, and molecular oxygen [3]. Upon light activation, the PS transitions to an excited state and transfers energy to surrounding oxygen molecules, generating reactive oxygen species (ROS) that can eradicate tumor cells. While PDT offers advantages like precision targeting and minimal invasiveness, its clinical translation faces significant hurdles, namely, poor tumor accumulation of hydrophobic PSs [4], hypoxia-induced treatment resistance [5], and limited immune activation [6].

To overcome these limitations, nanoencapsulation strategies for PSs are being actively developed [7–8]. Encapsulation of hydrophobic PSs in nanocarriers such as liposomes, polymeric nanoparticles, or micelles addresses solubility and stability, preventing aggregation [9]. Nanoparticles with sizes of 10–200 nm passively accumulate in tumor tissue via the enhanced permeability and retention (EPR) effect, driven by tumor vasculature characteristics [10]. This enhances delivery selectivity, reducing therapeutic doses and impact on healthy tissues. The nanoparticle shell isolates the PS, reducing dark toxicity and systemic phototoxicity [11–12].

Biomimetic nanoparticles (BNPs) represent a transformative novel approach for PS delivery [13]. By mimicking biological structures, such as cell membranes, proteins, or endogenous carriers, BNPs enhance PS delivery through improved biocompatibility, immune evasion, and active tumor targeting. Beyond delivery, BNPs address hypoxia [5], a critical barrier to PDT efficacy, through oxygen-carrying systems like hemoglobin-based nanostructures, which retain the oxygen-binding capacity of hemoglobin and can effectively transport and release oxygen within the hypoxic tumor microenvironment (TME). Furthermore, biomimetic platforms can synergize PDT with immunomodulation by promoting immunogenic cell death (ICD) and checkpoint inhibitor co-delivery [14–16]. By leveraging biomimicry, these systems seek to overcome biological barriers, increase treatment efficacy, and reduce side effects.

This review examines recent progress in the development of BNPs for cancer photodynamic therapy, with particular attention to their ability to target tumors, counteract hypoxia, and stimulate the immune system. We also explore their use in treating different types of cancer, the emergence of multifunctional platforms that combine imaging and therapy, and the challenges that remain for translating these technologies into clinical practice. By merging principles of biomimicry with nanotechnology, BNPs offer a promising new approach to more precise and effective cancer treatments tailored to individual patients.

### Overview of photodynamic therapy

PDT is a clinically established and continuously evolving treatment modality for various cancers and non-oncological conditions. Its therapeutic efficacy hinges on PSs, that is, specialized agents capable of absorbing light at specific wavelengths and transferring this energy to molecular oxygen or other substrates, generating cytotoxic ROS [17]. Historically, PDT relied on organic molecules, either naturally derived or synthetically produced, but since the early 21th century, advancements in nanotechnology have revealed the photosensitizing capabilities of nanostructured materials, significantly expanding the range of agents available for PDT applications [18].

**Figure 1:**
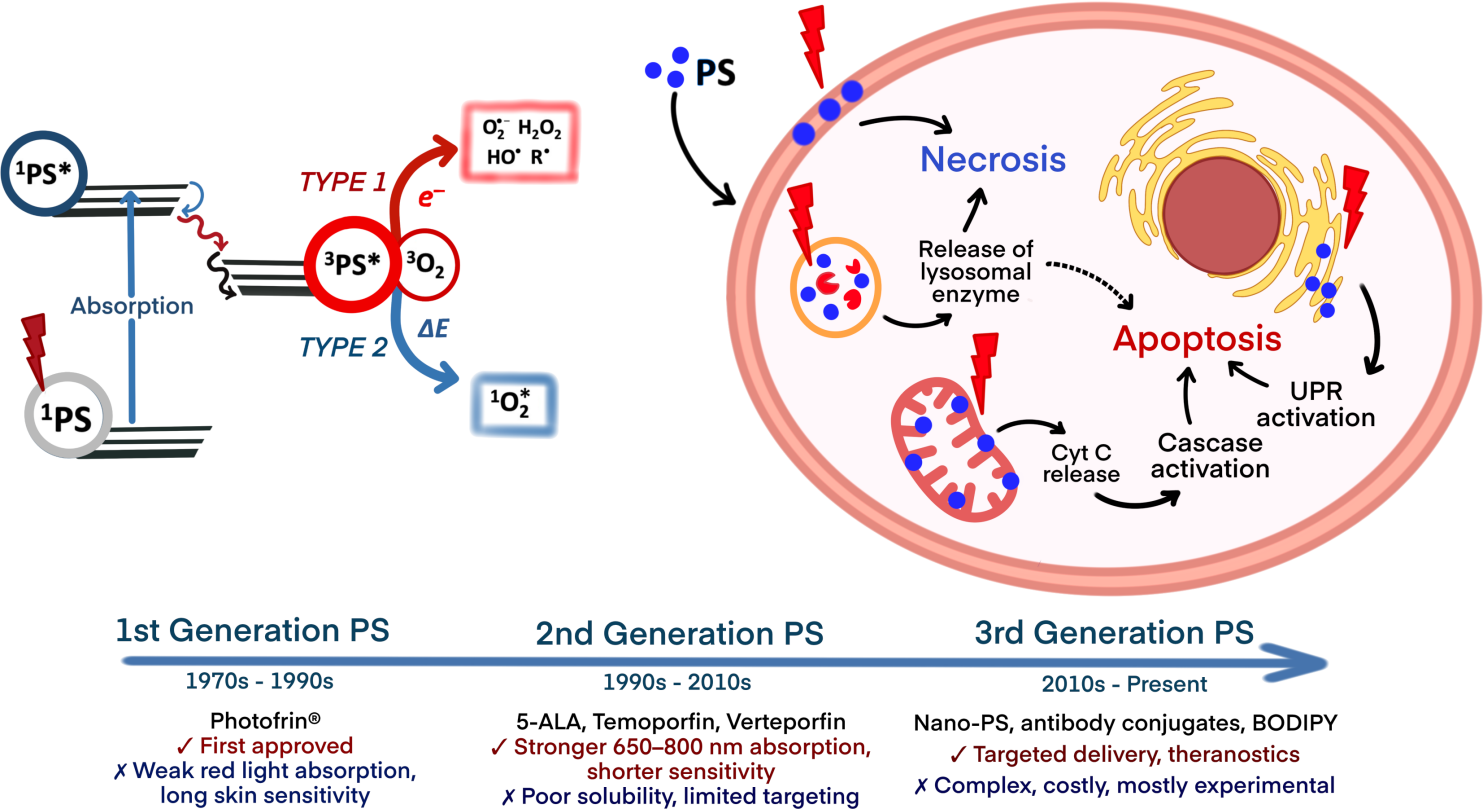
Photosensitizers in PDT: generations and types of PSs and their limitation, interaction of PSs with a cancer cell, mechanism of ROS generation by PSs, and induced death pathways (necrosis or apoptosis). The figure was created in BioRender. Syrocheva, A. (2026) https://biorender.com/mwpt7wl. This content is not subject to CC BY 4.0.

The photochemical pathway for ROS generation involves non-radiative relaxation of the excited PS, typically occurring through two primary mechanisms [19]. Type-I reactions involve electron transfer, producing radical species such as superoxide anions, while type-II reactions involve energy transfer, generating singlet oxygen, the primary cytotoxic agent in PDT (Figure 1). Although nanostructured PSs are often associated with type-I reactions and molecular PSs with type-II reactions, the generation of one ROS type frequently triggers cascades leading to others, making the distinction primarily about the predominant mechanism rather than exclusivity.

Efficacy and safety of PDT are largely determined by the PS’s properties [20]. An ideal PS must exhibit a high absorption coefficient in the near-infrared (NIR) range to enable deep tissue penetration, addressing a key PDT limitation. However, the use of longer wavelengths (>800 nm) is not practical due to their inefficiency in exciting an oxygen molecule from the triplet ground state to the excited singlet state [21]. A high quantum yield for ROS generation requires efficient excitation and minimal competing non-ROS-producing relaxation pathways. Low dark toxicity minimizes harm in the absence of light. High stability and low aggregation under physiological conditions are essential for maintaining photochemical activity [22]. A balanced hydrophilicity/lipophilicity ensures selective accumulation in target tissues, such as tumors via the EPR effect, and efficient cellular internalization. Low photobleaching sustains activity during treatment, while biocompatibility and biodegradability reduce adverse effects and enhance the therapeutic index. PSs are classified into generations, each reflecting progressive improvement (Figure 1).

The first generation of PSs included hematoporphyrin derivatives (e.g., Photofrin) [23]. The second generation of PSs encompasses purified compounds, including porphyrins [24], chlorins (e.g., chlorin e6 (Ce6) [25], mTHPC [26], bacteriochlorins [27], and phthalocyanines [28]), as well as non-porphyrinic structures like BODIPY dyes modified for enhanced ROS generation [29] and natural compounds such as hypericin and curcumin, primarily used in antimicrobial PDT [30]. Third-generation PSs focus on targeted delivery, achieved by conjugating second-generation PSs with carriers like antibodies, peptides, or nanoparticles to enhance specificity and reduce off-target effects [31–32].

The third generation of PSs should demonstrate minimal accumulation in healthy tissues and high selectivity toward pathological sites, thereby reducing side effects and increasing the therapeutic index. In this context, the use of nanocarriers for PS delivery gains particular importance, offering new opportunities to enhance both the selectivity and safety of PDT [33]. Currently, nanoparticles serve as versatile delivery system for a broad range of molecular therapeutics, including small molecules [34], RNA [35], plasmids [36], CRISPR/Cas systems [37], and other bioactive agents.

Several key advantages of nanocarriers in PDT can be highlighted: (i) improved solubility and stability of PSs, (ii) enhanced delivery through both passive and active targeting mechanisms, (iii) controlled and stimuli-responsive release profiles, (iv) overcoming of tumor hypoxia, (v) efficient light conversion, and (vi) the development of multifunctional platforms by combining PDT with other therapeutic modalities [38]. Additionally, nanoparticles enable theranostic applications [39] by facilitating tumor visualization through various imaging techniques [40], including fluorescence imaging [41], computed tomography [42], and magnetic resonance imaging (MRI) [43].

The development of nanobiotechnology in PDT opens new horizons for creating more effective and safer cancer treatments. Further research in this field is expected to drive the design of personalized PDT approaches tailored to the unique characteristics of individual patients and tumors, as well as to broaden the clinical application of PDT across a wide range of oncological diseases.

### Biomimetic nanoparticles: principles and platforms

#### The BNP concept: nature-inspired delivery

Advances in synthetic methods have enabled the fabrication of nanoparticles with a wide range of compositions, sizes, shapes, and surface properties [44–45]. These tunable characteristics make nanoparticles highly versatile for applications in disease diagnosis and sensing technologies. Despite the promising potential of nanomedicine, significant progress has been limited. This can be attributed to the low targeted accumulation of the nanocarriers at the desired site of action. For instance, in cancer therapy, the accumulation of nanoparticles within solid tumors typically does not exceed 1% of the administered dose [46]. The limited progress in achieving higher carrier targeted accumulation can be attributed to two main factors: (i) rapid clearance of the nanoparticles from the body, that is, nanoparticles are cleared from the systemic circulation by the organs and the cells of the mononuclear phagocyte system (MPS), primarily the liver and spleen, and (ii) ineffective overcoming of biological barriers, that is, nanoparticles have to overcome numerous biological barriers to reach the target tissue, including extravasation from the vasculature, penetration through the interstitial space, and cellular internalization at the target site [47]. Conferring the carriers with a biomimetic shell that mimics the functions of biological cells potentially increases their circulation time and targeting properties, while significantly reducing the body’s immune response [48]. BNPs utilizing a cell membrane to coat a synthetic core were reported in 2011. These nanoparticles consisted of a poly(lactic-*co*-glycolic) acid (PLGA) core coated with an outer layer derived from red blood cell (RBC) membranes [49]. It was demonstrated that the presence of this biomimetic shell increased more than two times the circulation time of the carriers in the bloodstream compared to uncoated PEGylated (polyethylene glycol-coated) nanoparticles, representing the gold standard approach to nanoparticle circulation time. Since then, a wide variety of cell phenotypes have been explored as sources for biomimetic membranes to coat nanoparticles such that inherent biological functions and properties of specific cells could be exploited to enhance the performance and versatility of BNPs. According to some authors, biomimetic systems comprising proteins from cells to mimic their functions belong to the third generation of nanodelivery systems [50]. The first generation of particles was based on surface modifications to reduce the interactions with immune cells and to increase biocompatibility. Among them, biocompatible polymers like PEG and PPE have been widely used [51]. The second generation of nanocarriers harnessed surface functionalization with antibodies, peptides, and aptamers to increase the targeting of pathogenic tissues and cells by interacting with the receptors expressed on the surface of the target cells [52]. In contrast, BNPs are designed to imitate the biological identity of cells through the corresponding surface proteins and clusters of biological molecules; these modifications enable the actions of aforementioned technologies and provide the particles with new functions.

#### Limitations of biomimetic nanoparticles

The clinical translation of BNPs is primarily hindered by challenges related to large-scale production, reproducibility, and quality control. The multistep fabrication process, including extraction of cellular membranes and coating of nanoparticle cores, requires careful optimization to ensure stability and consistency [53].

Cell lysis is essential for the extraction of biomimetic membranes; the membrane composition is directly influenced by the severity of the lysis method. Common techniques include (i) ultrasound, that is, membrane rupture via pressure waves (20–50 kHz), requiring precise control to prevent heat damage [54]; (ii) freeze–thaw cycles, that is, cell disruption via narrow pores (enhanced at low temperatures) [55]; (iii) mechanical shearing, that is, cell disruption via narrow pores (enhanced at low temperatures) [56]; and (iv) osmotic pressure, that is, hypotonic solutions are used to induce swelling and rupture [53].

Lysis buffer selection (containing membrane disruptors and protease inhibitors) depends on cell type and target protein localization. For example, cancer cells and macrophages respond better to hypotonic protocols [57], and harsh homogenization suits cytomembrane protein extraction (above 30% cell composition).

Various methods exist for coating synthetic nanoparticle cores with biomimetic shells, each with unique advantages and limitations. A crucial factor is ensuring the correct “right-side-out” orientation of membrane proteins, which preserves system stability and enables proper interaction with target cells [58]. Common techniques include (i) physical co-extrusion, where nanoparticles and membranes are forced through porous membranes to form uniform coatings; (ii) sonication, which uses ultrasonic energy to induce membrane fusion and often favors correct protein orientation; and (iii) electroporation, which creates temporary pores in membranes to facilitate coating while preserving protein integrity. Electroporation is simpler, more versatile, and scalable, especially when combined with microfluidic devices that allow for precise control over coating conditions, improving uniformity and reproducibility [59]. Tailoring and combining these approaches enables the design of BNPs with desired functionalities, though challenges remain in standardizing protocols and scaling up production.

Other significant limitations include nonuniform coating, difficulties in purification, and maintaining sterility, especially for BNPs derived from bacterial membranes [60]. Heterogeneous coverage can affect nanoparticle stability and drug delivery efficiency, while purification processes must effectively separate fully coated particles from impurities. For bacterial membrane-based BNPs, thorough detoxification is essential to eliminate toxic components without compromising immunogenic properties [61].

Finally, the biomimetic coating can influence drug release profiles, potentially hindering the controlled release of therapeutic agents [62]. Optimizing these systems requires careful consideration of coating thickness, core material, and release mechanisms. Addressing these challenges through standardized production protocols, improved characterization, and enhanced biocompatibility will be critical to advancing BNPs toward clinical application.

Biomimetic nanocarriers, designed to replicate structure and functions of biological cells, present promising advantages for drug delivery and therapeutic applications. Nonetheless, they face notable challenges that require thorough investigation. Key areas of focus include developing standardized, scalable manufacturing techniques, elucidating their interactions with cells and mechanisms for crossing physiological barriers, enhancing stability for controlled drug release, and improving biocompatibility to ensure clinical safety. Tackling these challenges is essential to optimize biomimetic nanocarriers for better efficacy and successful translation into clinical practice.

#### Crossing of biological barriers

BNPs face a series of biological barriers upon administration, which significantly influence their therapeutic and diagnostic effectiveness [63]. One of the first challenges is opsonization, where plasma proteins form a “protein corona” [64] on the nanoparticle surface, often reducing targeting specificity and biocompatibility. Coating nanoparticles with cell membranes, such as those from RBCs or leukocytes, can minimize protein corona formation, preserve targeting ligands, and extend circulation time by evading immune clearance. This strategy leverages natural “self-markers” like CD47 to inhibit phagocytosis and enhance passive tumor accumulation via the EPR effect [65].

Immune clearance by the MPS, primarily in liver and spleen, is another major hurdle. Biomimetic coatings help nanoparticles avoid rapid removal from the bloodstream, as demonstrated by prolonged circulation and increased tumor targeting in systems coated with erythrocyte or stem cell membranes [66]. Such coatings can also facilitate homotypic targeting, where nanoparticles preferentially bind to and are internalized by cancer cells of the same origin, further improving specificity and reducing off-target effects.

The behavior of BNPs in the bloodstream is also shaped by hemodynamics and vessel wall interactions. Platelet-mimicking nanoparticles, for example, can adhere to vascular injury sites and inflamed tissues, enhancing accumulation at target areas and even supporting hemostasis. These properties are particularly valuable for treating inflammatory diseases and targeting tumors.

Crossing the blood–brain barrier (BBB) remains a significant challenge for nanomedicine. BNPs coated with membranes from cells capable of BBB penetration, such as neutrophils or cancer cells, show improved brain delivery [67]. Additional modifications with targeting peptides can further enhance BBB crossing, opening new possibilities for treating neurological diseases.

Finally, successful therapy depends on efficient cellular uptake and drug release. Biomimetic shells can increase nanoparticle internalization through receptor-mediated endocytosis and homotypic binding, as well as improve colloidal stability. Strategies such as incorporating pH-sensitive components or lysosomal escape mechanisms further enable controlled drug release within target cells. Despite these advances, optimizing BNP design to overcome all biological barriers remains an active area of research essential for clinical translation.

#### BNP functionalization

The development of biomimetic drug delivery systems has advanced rapidly, with multifunctional platforms emerging as a key focus [68–69]. By leveraging the natural properties of cell membranes, such as immune evasion, prolonged circulation, and specific targeting, BNPs can be further enhanced through surface modifications to improve their therapeutic performance. These modifications enable BNPs to address complex challenges in drug delivery, including targeted transport, controlled release, and overcoming biological barriers.

One promising strategy is the creation of hybrid biomimetic coatings, where membranes from multiple cell types are fused to combine their unique functionalities. For example, NPs coated with a mix of RBC, cancer cell, and immune cell membranes can simultaneously evade the immune system, target tumors, and deliver drugs to metastatic sites. This approach has demonstrated improved blood circulation, enhanced tumor targeting, and increased therapeutic efficacy in preclinical models. However, the process of membrane fusion and the need for additional purification steps add complexity to manufacturing [70].

Genetic modification of source cells offers another avenue for BNP functionalization. By engineering cells to express specific targeting proteins or receptors before membrane extraction, it is possible to produce BNPs with highly selective targeting capabilities. For instance, membranes from CAR-T cells or stem cells engineered to overexpress homing receptors can be used to create BNPs that efficiently recognize and bind to tumor cells or inflamed tissues, minimizing off-target effects and systemic toxicity [71–72].

Post-synthesis surface modification is also widely used to enhance BNP functionality [73–75]. Non-covalent methods, such as lipid insertion, allow for the integration of targeting ligands, peptides, or imaging agents into the membrane without disrupting protein activity. Covalent modifications, including the attachment of antibodies or peptides via amide or thiol–maleimide linkages, provide stable and specific functionalization but may risk altering membrane protein function if not carefully controlled. Enzymatic approaches can offer greater specificity but require additional steps and conditions.

**Figure 2:**
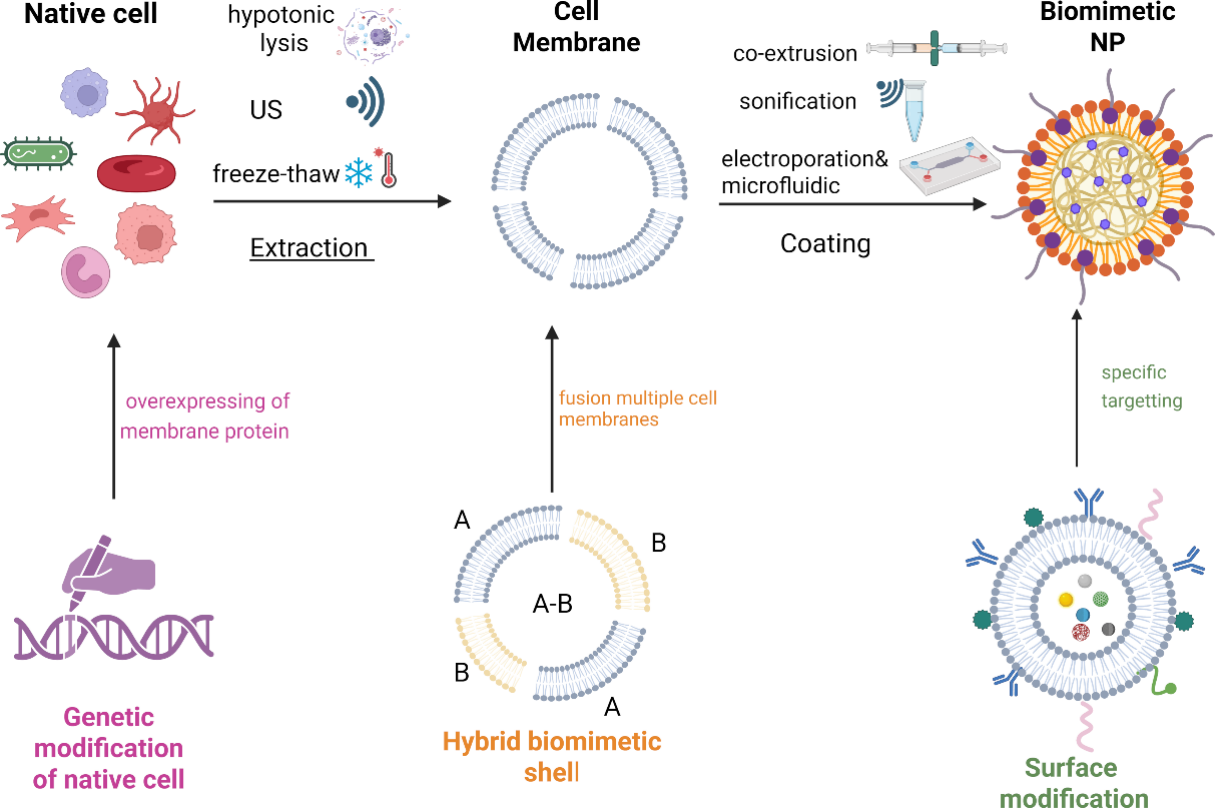
Processes for fabricating and functionalizing biomimetic nanoparticles (BNPs), highlighting key methodologies for cell membrane extraction, coating, and surface engineering to achieve enhanced tumor targeting and therapeutic performance in photodynamic therapy. The figure was created in BioRender. Syrocheva, A. (2026) https://biorender.com/ksvnysj. This content is not subject to CC BY 4.0.

These advanced BNPs can be engineered to respond to specific stimuli, such as pH, enzymes, or external triggers like light, enabling controlled drug release at the target site [76]. For example, pH-sensitive lipids or peptides can be incorporated to accelerate drug release in the acidic tumor microenvironment. Additionally, BNPs can be loaded with imaging agents or therapeutic enzymes to facilitate tumor visualization and penetration, further expanding their utility in cancer therapy. Targeting strategies can be further refined by integrating ligands such as arginylglycylaspartic acid (RGD) peptides, antibodies, or aptamers onto the BNP surface, enhancing their ability to recognize and bind to specific cell markers [77]. Incorporating imaging dyes or contrast agents into the BNP membrane also enables real-time tracking and monitoring of drug delivery in vivo. The main stages of biomimetic nanoparticle fabrication and subsequent surface modification are summarized in Figure 2.

### BNPs for enhanced delivery and specificity in PDT

BNPs achieve spatial specificity within the TME through a hierarchical targeting strategy, combining passive accumulation mechanisms with innate or engineered active targeting capabilities. This multilevel approach significantly enhances the delivery efficiency of PSs compared to conventional nanocarriers [78].

Passive targeting primarily exploits EPR effect, a pathophysiological feature of many solid tumors characterized by leaky vasculature and impaired lymphatic drainage [79–80]. BNPs amplify passive accumulation through their extended circulation time, a direct consequence of immune evasion conferred by their biomimetic coatings [81]. Prolonged systemic exposure increases opportunities for extravasation through tumor vasculature. For instance, erythrocyte membrane-coated BNPs consistently demonstrate 1.5- to 2.0-fold higher tumor accumulation than their uncoated counterparts with identical cores, attributable solely to superior circulation half-life and EPR potentiation [82–84]. While EPR is a universal mechanism for nanocarriers, the significant enhancement provided by biomimetic coatings, particularly erythrocyte and mesenchymal stem cell membranes, represents a critical advantage over synthetic platforms, enabling greater passive PS deposition within tumors [85].

Active targeting utilizes specific molecular interactions to enhance tumor cell binding and internalization. BNPs employ both innate (source cell-derived) and engineered strategies to achieve this. Innate active targeting exploits the inherent biological tropism preserved on the source cell membrane, requiring no additional modification [86]. As exemplified by mesenchymal stem cell (MSC) membrane-coated BNPs, this system can utilize the natural homing capability of the source cells (e.g., MSCs migrating towards TME chemokine gradients like SDF-1α and CCL2) [87]. This conserved tropism results in significantly higher tumor accumulation compared to untargeted controls [88].

The biological specificity of different cell types is determined by proteins present on their plasma membranes, which not only serve structural roles but also mediate cell–cell adhesion, either homotypic or heterotypic, depending on molecular affinity. The primary mechanisms underlying homotypic binding involve various classes of adhesion proteins, such as integrins, selectins, cadherins, and members of the immunoglobulin superfamily (IgSF). Integrins typically interact with the extracellular matrix, whereas selectins, cadherins, and IgSF proteins are primarily responsible for cell–cell adhesion [89]. The cytoplasmic and extracellular domains of these proteins confer distinct physicochemical properties that influence cellular interactions and tissue organization. Given the crucial role of integrins in tumor progression, there is significant interest in their use not only as markers of malignant potential but also as targets for precision therapies.

**Figure 3:**
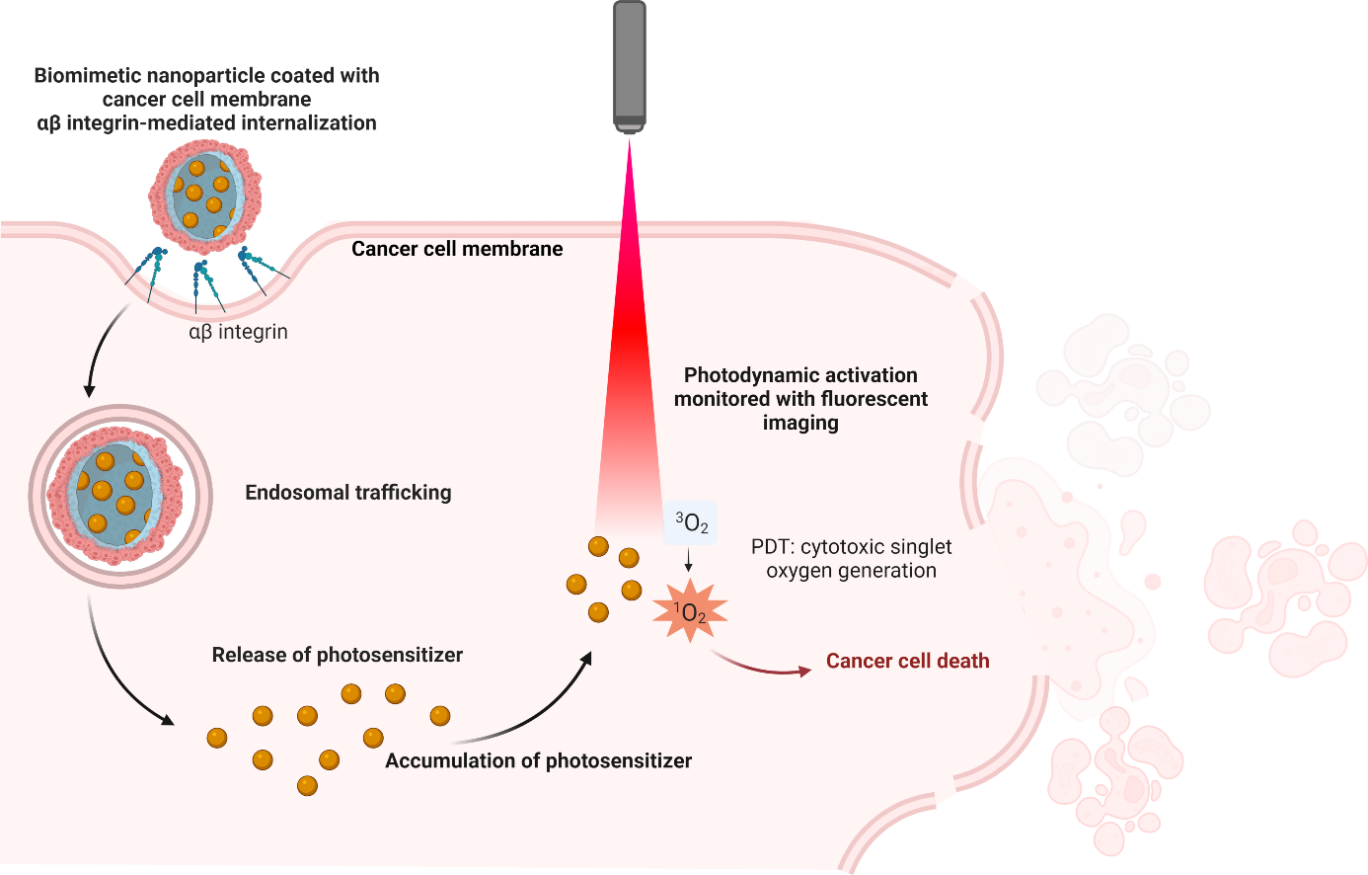
Selective targeting and internalization via homotypic binding and receptor-mediated endocytosis of biomimetic nanoparticles coated with cancer cell membranes, enabling enhanced photosensitizer accumulation and reactive oxygen species generation for improved photodynamic therapy. The figure was created in BioRender. Syrocheva, A. (2026) https://biorender.com/jjvlem9. This content is not subject to CC BY 4.0.

One of the most extensively studied integrins, αVβ3, is a transmembrane receptor for extracellular matrix proteins that binds cyclic RGD peptides with high affinity and interacts with SRC oncogene to promote lymph node metastasis. For example, RGD-coated BNPs enhanced doxorubicin (DOX) uptake by 1.4 times in αVβ3-positive U-87 MG glioblastoma cells compared to αVβ3-negative HeLa cells [90]. In urological cancers, integrin α5β1 is upregulated, facilitating cell adhesion to fibronectin; PHSCN peptide-targeted BNPs have shown efficacy in inhibiting prostate cancer metastasis in preclinical and clinical settings [91]. High malignancy cancer cells display reduced homotypic adhesion but increased heterotypic adhesion to endothelial cells, mediated by molecules such as IL6R [92], CXCR4 [93], and EPCAM [94], which are targeted via peptide-functionalized BNPs to inhibit metastasis. Additionally, NGR peptides targeting aminopeptidase N have improved photoinduced activity of chlorin e6 in fibrosarcoma cells [95]; dual peptide modification of PLGA nanoparticles enhanced brain targeting in glioma models [96]. Integrin expression varies across tumor types, influencing homotypic targeting strategies [89,97]. A membrane coverage of 50% and more enables cellular uptake of individual nanoparticles, while lower coverage requires aggregation for internalization [60]. Using cancer cell membranes (CCMs) with high coverage on BNPs ensures selective targeting through integrin-mediated homotypic binding, promoting efficient therapeutic delivery (Figure 3). Relevant proteins enabling homotypic targeting via BNPs are listed in Table 1.

**Table 1:** A list of proteins that can be incorporated into biomimetic nanoparticles to facilitate targeted delivery via homotypic binding.

Protein	Cell lines	Cancer type	Application in drug delivery	Ref

α- and β-integrins	4T1, BT-20, B16, C4-2B	breast cancer, breast carcinoma, melanoma, prostate cancer, bone metastases	In biomimetic nanoparticles, proteins and peptides containing the RGD (arginine-glycine-aspartate) motif are most often used for targeted binding to α- and β-integrins. The main proteins used in such systems are fibronectin, vitronectin, and laminin, as well as synthetic RGD-based peptides (for example, cyclic RGD peptides). The use of fibronectin in the coating of nanoparticles loaded with doxirubicin, tannic acid, and iron contributed to the targeted therapy of the B16 tumor with high expression of integrin a5b3. Intracellular absorption of nanoparticles was confirmed by confocal laser scanning microscopy. For the treatment of prostate cancer C4-2B, poly(lactic-*co*-glycolic acid) nanoparticles coated with cancer cell membranes containing integrin β3, a subunit of integrin α5ß3, were used. The resulting nanoparticles demonstrated a fourfold increase in absorption in C4-2B cells compared to uncoated nanoparticles, which was measured using flow cytometry.	[98–100]
E-cadherin and N-cadherin	4T1 cells	breast cancer, lung metastases	4T1 cancer cell membrane-coated paclitaxel nanoparticles showed 3.3 times and 2.5 times higher accumulation in primary breast tumors and lung metastases versus uncoated NPs. Negative controls (RBC-coated and liposome-coated NPs) had lower uptake. E- and N-cadherins mediated binding.	[101]
P-selectin	H22 cells	hepatoma	Platelet membrane-coated biomimetic nanoparticles loaded with bufalin targeted hepatoma via P-selectin–CD44 interaction, enhancing uptake and inhibiting tumor growth.	[102]
E-selectin	A549, Hs 578T	circulating tumor cells (various)	Surfactant–nanotube biomimetic complexes with E-selectin facilitated capture of circulating A549 and Hs 578T carcinoma cells, confirmed by flow cytometry.	[103]
galectin-1 and galectin-3	4T1 cells	breast cancer	biomimetic nanoparticles containing calcium phosphate and STING Mn2+ agonist, Cypate photosensitizer, and indolamine 2,3-dioxygenase 1 (IDO1); the inhibitor was coated with galactose to act on galectin-1 and galectin-3 receptors; after intravenous administration to mice, this system demonstrated high accumulation in 4T1 tumors, which ensured accurate diagnosis and effective elimination.	[104]

Early studies on BNPs often employed simple coatings derived from CCMs. This approach effectively camouflaged the nanoparticles by conferring the properties of “self” cells, thereby enhancing biocompatibility and reducing neutrophil-mediated immune clearance. For instance, BNPs loaded with DOX and coated with glioblastoma cell membranes were employed as a targeted therapy for malignant tumors. The presence of surface adhesion molecules, including N-cadherin, galectin-3, and epithelial cell adhesion molecules, facilitated enhanced uptake of the drug in the human glioblastoma U87 MG cell line. This resulted in an 8.5-fold increase in cellular internalization compared to uncoated nanoparticles [105]. However, over time, it became evident that such simple membrane coatings have limited functional capabilities. This realization has driven the development of additional surface modifications aimed at improving targeting specificity to particular cells or tissues, enhancing stability in the bloodstream, controlling drug release kinetics, and tailoring nanoparticle systems to meet diverse clinical requirements. Recent advances in this field have demonstrated the remarkable efficacy of homophilic targeting. Specifically, the involvement of CD47 from lung CCM has been shown to facilitate evasion of phagocytosis, while N-cadherin, galectin-3, CD44, and CD326 mediate homotypic binding to cancer cells; this resulted in a threefold increase in the accumulation of PLGA nanoparticles within the tumor [106]. A notable study aimed to evaluate the translational potential of biomimetic delivery systems for precise tumor therapy. Gold@carbon nanoparticles were coated with proteins derived from different cell types, including a squamous cell carcinoma cell line and patient-derived cells. To address tumor heterogeneity, these systems were tested across various xenograft models, namely, cell line-derived subcutaneous xenografts, orthotopic tongue xenografts, immunocompetent primary and distant tumor models, and patient-derived xenografts. In all models, the biomimetic coating enhanced nanoparticle accumulation within tumors and improved therapeutic efficacy. The authors emphasize the importance of such comprehensive evaluations to facilitate the translation of these findings into clinical practice [107].

**Figure 4:**
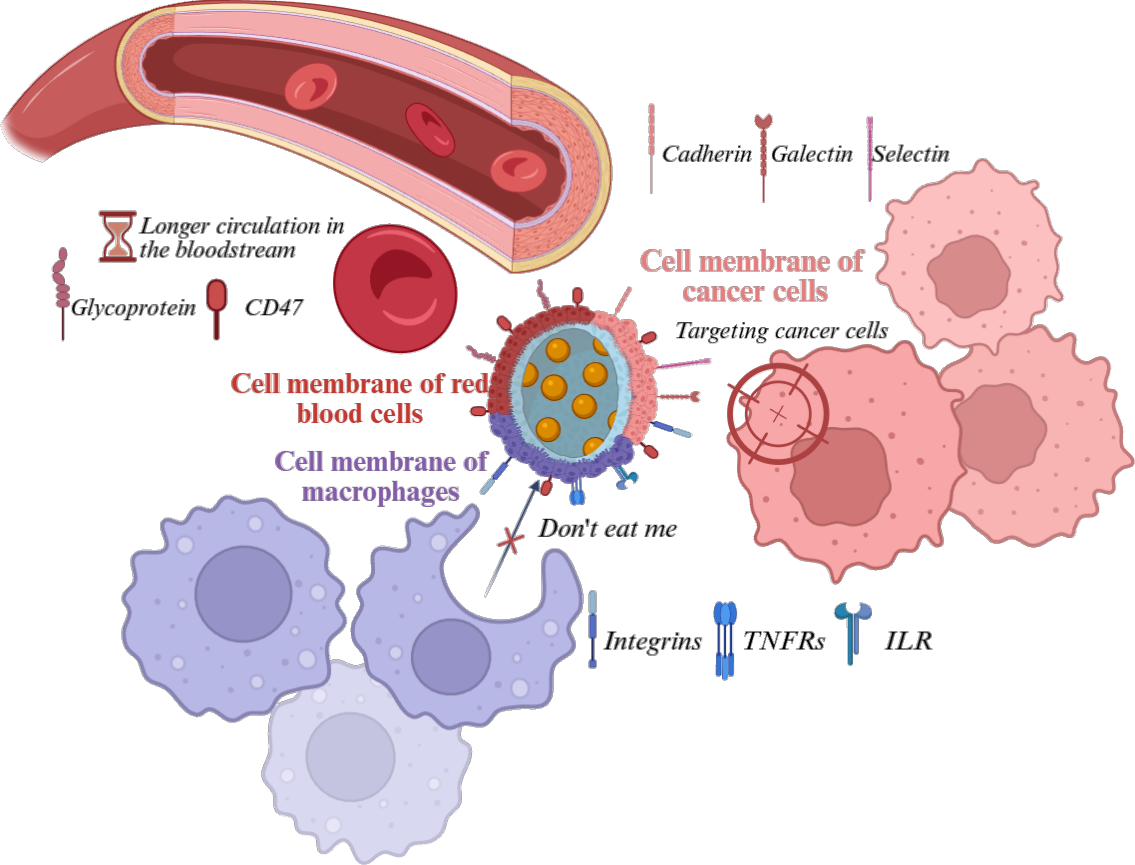
Modifications of biomimetic nanoparticle coatings using erythrocyte, macrophage, and cancer cell membranes, conferring unique functional properties including prolonged systemic circulation, immune response evasion, and homotypic tumor targeting for enhanced photodynamic therapy. The figure was created in BioRender. Syrocheva, A. (2026) https://biorender.com/ffuwoj1. This content is not subject to CC BY 4.0.

A prominent trend in drug delivery is the creation of multifunctional, stable, and biocompatible platforms with tunable biological activity, often achieved by using hybrid membrane coatings that combine proteins from multiple cell phenotypes, thereby enhancing their therapeutic and diagnostic versatility. The concept of hybrid membrane coating was first introduced in 2017 [108], where PLGA nanoparticles were coated with combined erythrocyte and platelet membranes, leveraging their unique functions to evade immune clearance and target inflammatory sites. Since then, an increasing variety of differentiated cell membranes have been combined to modify biomimetic nanoparticle surfaces, such as cancer cell–erythrocyte [108] and macrophage-–cancer cell [109] hybrids (Figure 4). For combinational therapy of triple-negative breast cancer, BNPs based on tumor cell membranes and PLGA loaded with the cytolytic peptide melittin and the photosensitizer mTHPC have been developed. In vitro and in vivo studies demonstrated that these nanoparticles mitigated the acute toxicity of melittin and enhanced tumor accumulation of mTHPC in 4T1 tumor-bearing mice, while facilitating accelerated melittin release due to disruption of the membrane integrity [110].

Similar to other nanodelivery systems, a wide variety of surface functionalization strategies have emerged to enhance targeting efficiency in BNPs. In a 2022 study, a sophisticated biomimetic system was developed comprising paclitaxel nanocrystals coated with SKBR-3 cell membranes. The membrane coating was further functionalized with Herceptin, a monoclonal antibody that selectively binds to HER2 receptors, rendering the platform a promising candidate for the treatment of HER2-positive breast cancer [111]. In vivo experiments demonstrated homotypic targeting, significantly enhanced cellular uptake compared to uncoated nanocrystals, and confirmed the system’s ability to induce apoptosis in HER2-positive breast cancer cells. Another study reported the development of a multifunctional biomimetic system coated with erythrocyte membranes for the co-delivery of chlorin e6 and DOX to enhance synergistic targeted tumor therapy. This system exhibited high targeting efficiency toward HepG2 cells via folic acid receptor-mediated endocytosis, followed by effective tumor eradication through singlet oxygen generation. Modification with erythrocyte membranes also conferred prolonged circulation time to the nanoparticles, attributed to the presence of the CD47 molecule [112].

An alternative approach to the functionalization of biomimetic coatings is based on genetic engineering. In a recent study employing this strategy, the cell membrane from a genetically engineered HEK-293 cell line expressing synthetic SpyCatcher proteins was used to coat NPs, enabling targeted binding to both tumor cells and viral particles. Treatment with these nanoparticles in a mouse model of ovarian cancer suppressed tumor growth and improved survival outcomes [113]. For the therapy of uveal melanoma, BNPs coated with recombinant low-density lipoproteins were developed to deliver the PS verteporfin and the vascular-normalizing agent dexamethasone. Following intravenous administration, these nanoparticles selectively accumulated in tumor tissue; verteporfin generates ROS, resulting in tumor cell death, while the ROS also facilitated rapid release of dexamethasone, which inhibited neovascularization in the tumor microenvironment. Thus, the ability to program specific protein sequences allows for their precise display on the nanoparticle surface in physiologically relevant conformations and orientations, thereby enhancing their functional activity in binding to cellular receptors.

A key emerging trend in such delivery systems is the development of smart platforms that respond to external stimuli, enabling remote-controlled release of therapeutic payloads. A significant advancement came from Wang’s group in 2023 [114], who developed “adaptive” CCM-coated nanoparticles that could dynamically alter their surface properties in response to the TME. By incorporating pH-sensitive linkers between the membrane coating and targeting ligands, these nanoparticles maintained their stealth properties during circulation but exposed their homophilic binding sites specifically within the acidic tumor environment, resulting in a 2.5-fold increase in tumor-specific uptake compared to conventional membrane-coated systems.

#### Main classes of biomimetic nanoplatforms for photosensitizer delivery

Exosome-based platforms utilize naturally secreted extracellular vesicles (40–100 nm) possessing a lipid bilayer and a unique surface protein profile derived from various cell types (e.g., dendritic cells, T cells, and macrophages) [86,115–116]. Their endogenous role in intercellular communication confers low immunogenicity and high biocompatibility, facilitating evasion of immune clearance [117]. A key advantage is their ability to penetrate biological barriers, including the BBB [118], enabling access to challenging tumor sites like glioblastoma [119]. The surface-exposed proteins support inherent cell-specific interactions, which can be augmented by engineering targeting ligands for improved tumor specificity [120]. In PDT, exosomes effectively deliver PSs like Ce6, demonstrating enhanced tumor accumulation and photodynamic efficacy compared to free PSs [121]. Challenges include complex isolation procedures, heterogeneity, scalability limitations, and potential loss of membrane protein activity during processing [122].

CCM-coated platforms involve cloaking synthetic nanoparticle cores (e.g., PLGA, silica, and metal-organic frameworks (MOFs)) with membranes derived from tumor cells [123]. This biomimetic strategy primarily exploits homotypic targeting; the CCM retains adhesion molecules (e.g., galectin-3 or T-antigen binding proteins) enabling preferential binding and deep penetration into homologous tumor tissues. CCM-coated NPs have successfully delivered diverse PSs (e.g., Ce6 [124–125], PCN-224 [126], and IR-780 [127–128]), often in combination therapies (e.g., chemo-PDT and immune checkpoint blockade [129]), achieving high tumor accumulation and potent PDT responses in preclinical models. Critical limitations include the inherent specificity of homotypic targeting to the parent cancer cell type, restricting broad applicability, and scalability challenges in membrane production and fusion.

RBC membrane-coated platforms employ the readily available membranes of RBCs to encapsulate nanoparticle cores [130]. Their primary advantages are their exceptional ability to evade the immune system and their long circulation half-life, which exceeds 100 days in vivo for native RBCs [84]. These properties have popularized two major strategies, that is, using RBC membranes as a biomimetic shell for nanoparticles [131] and directly targeting circulating RBCs in vivo for drug delivery [132]. This is largely mediated by CD47 expression on the RBC membrane, which delivers a potent “do not eat me” signal by binding to SIRP-α on phagocytes [133]. This inherent property ensures high biocompatibility and minimal toxicity. While lacking innate tumor targeting, RBC-coated NPs can be effectively functionalized with ligands (e.g., folate [83,134] or peptides like RGD [135–136]) to achieve active targeting, enhancing accumulation at tumor or infection sites. These platforms efficiently deliver PSs (e.g., Ce6 [137], TPC [138], MC540 [83], indocyanine green (ICG) [139], or IR-780 [140]), leveraging the EPR effect, long circulation times, and potential ligand-mediated targeting to improve tumor PS levels, thereby enhancing PDT efficacy and enabling combination strategies (e.g., with anti-PD-1 immunotherapy [141]). The need for additional functionalization for specific targeting and potential batch variability in membrane extraction represent key considerations.

MSC membrane-coated platforms utilize membranes derived from stem cells to cloak nanoparticle cores [142]. A defining characteristic is their ability to mimic the tumor tropism of native MSCs. This is achieved through the retention of chemokine receptors (e.g., CXCR4) on the MSC membrane, which mediate specific adhesion to ligands (e.g., SDF-1α) prevalent on the endothelium within TME [143]. This property facilitates active migration towards tumor and metastatic sites. Additionally, MSC membranes confer low immunogenicity and reduced MPS clearance, enhancing overall biocompatibility and tumor penetration. MSC-coated NPs have been employed to deliver PSs such as Ce6 [144], ZnPc, and MC540 [145], demonstrating improved tumor accumulation and cellular uptake compared to non-biomimetic counterparts. While the tropism is advantageous, the precise molecular mechanisms governing MSC homing require further elucidation, and scalability/reproducibility of MSC culture and membrane isolation remain practical challenges.

Immune cell membrane-based platforms incorporate membranes from various immune cells (e.g., macrophages, neutrophils, dendritic cells (DCs), T cells, and natural killer (NK) cells) onto nanoparticle cores or utilize derived extracellular vesicles [53]. Immune cell membrane-based BNPs represent a breakthrough platform that seamlessly integrates PDT with immunotherapy. The membranes retain source cell-specific functionalities: (i) Macrophage membranes exhibit tropism towards inflammatory sites and tumors via chemokine receptors (e.g., CCR2) and adhesion molecules. Pre-polarization to an M1 phenotype ex vivo can further equip these nanoparticles to reprogram the immunosuppressive TME [146–147]. (ii) Neutrophil membranes inherently target inflamed endothelium and tumor sites; facilitate crossing vascular barriers [148]. (iii) DC membranes possess inherent homing to lymph nodes and carry tumor-associated antigens, promoting antigen presentation and T-cell activation when combined with PDT-induced ICD [149]. (iv) T-cell membranes (including CAR-T) enable specific antigen recognition via TCR or CAR [150–152]. (v) NK cell membranes inherit natural cytotoxicity receptors (e.g., NKG2D and NKp30) for innate tumor recognition and killing activation [153]. (vi) Platelet-derived membranes exhibit natural adhesion to damaged vasculature (common in tumors) and circulating tumor cells (CTCs); they also express CD47 for extended circulation and modulate immune cell interactions and inflammation [154].

These properties allow for deep tumor penetration and direct interaction with immune components within the TME. Consequently, immune cell BNPs can deliver PSs while simultaneously modulating the immune landscape; for instance, they promote ICD via PDT and concurrently deliver checkpoint inhibitors (e.g., anti-PD-1/PD-L1) or immunomodulators (e.g., R848). This potent combination therapy approach is explored further in the context of immune modulation. Challenges include the complexity and variability of immune cell membrane isolation, potential for immune rejection depending on allogenicity, reproducibility, and scalability. Hybrid platforms represent an advanced strategy where membranes from two or more distinct cell types (e.g., RBC + cancer cell, RBC + immune cell, and cancer cell + macrophage) are fused to create a single biomimetic coating [155]. This approach combines the unique advantages of each parental membrane.

#### PS loading into biomimetic nanocarriers

Efficient encapsulation of PSs into BNPs is a pivotal step that directly influences the overall therapeutic outcome of PDT. The success of PDT hinges not only on the intrinsic photochemical properties of the PS but also on its effective delivery to the tumor site, stability during circulation, bioavailability within the TME, and ability to be activated by light to generate ROS. BNPs, by virtue of their biological surface functionalities and synthetic nanoscale architecture, offer sophisticated platforms that maximize these criteria. However, the process of loading PS into such carriers must be carefully optimized to maintain the delicate balance between high loading capacity, preserved photophysical activity, controlled release, and targeting specificity. Several advanced strategies have been developed and tailored to integrate PSs into BNPs. These approaches take into consideration the hydrophobic nature of the PS, the chemical functionalities available for conjugation, and the physicochemical characteristics of the carrier. For instance, encapsulation techniques must ensure that PS molecules remain stably entrapped within the nanoparticle core to prevent premature leakage during blood circulation while enabling efficient release or activation within the tumor site.

A widely adopted approach is to first encapsulate hydrophobic PS molecules into synthetic nanoparticle cores. These stable PS-loaded cores are subsequently cloaked with natural cell membranes, imparting immune evasion, prolonged circulation, and targeting. Ce6 was loaded to methoxy PEG-PLGA NPs via microfluidic flow, covered with RBC membrane and modified with folic acid. The loading efficiency was 20%, and coating with the biomimetic membrane reduced the initial burst release during the first 4 h while sustaining gradual release over 48 h. The biomimetic coating increased the rate of cancer cell apoptosis induced by the photodynamic effect by 50% and prolonged the circulation time of the nanoparticles in the bloodstream [112].

BNPs composed of materials such as melanin or polydopamine exploit strong π–π stacking interactions with aromatic PSs like Ce6 to achieve high loading capacity. For instance, melanin-based nanoparticles loaded with Ce6 (loading efficiency ≈ 30%) showed enhanced biocompatibility and water solubility, with synergistic photodynamic and photothermal effects under imaging guidance [156].

Another strategy involves chemically attaching porphyrin-based PSs bearing conjugatable functional groups (e.g., carboxyl) onto the surface of bioinspired nanoparticles such as noble metal nanoparticles (Ag and Au). Stable ester bond formation secures the PS to the nanocarrier surface, offering hydrophilicity, biocompatibility, and enhanced biological delivery. Loading efficiencies of ≈16% have been reported for synthesized porphyrin PS and bioinspired metal nanoparticles (rich in –OH groups), suitable for dual PDT and photothermal therapy (PTT) [157].

Some biomimetic platforms enable co-loading of the PS with other agents to boost therapeutic outcomes. For example, DOX was preloaded into RBC nanovesicles while Ce6 was encapsulated in polymeric cores coated with RBC membranes [112]. Doxorubicin and ICG were loaded to BNPs with CCMs and in vivo demonstrated high tumor ablation with low systemic toxicity. This multifunctional system combines chemotherapy and PDT, together with a number of diagnosis tools [158].

**Figure 5:**
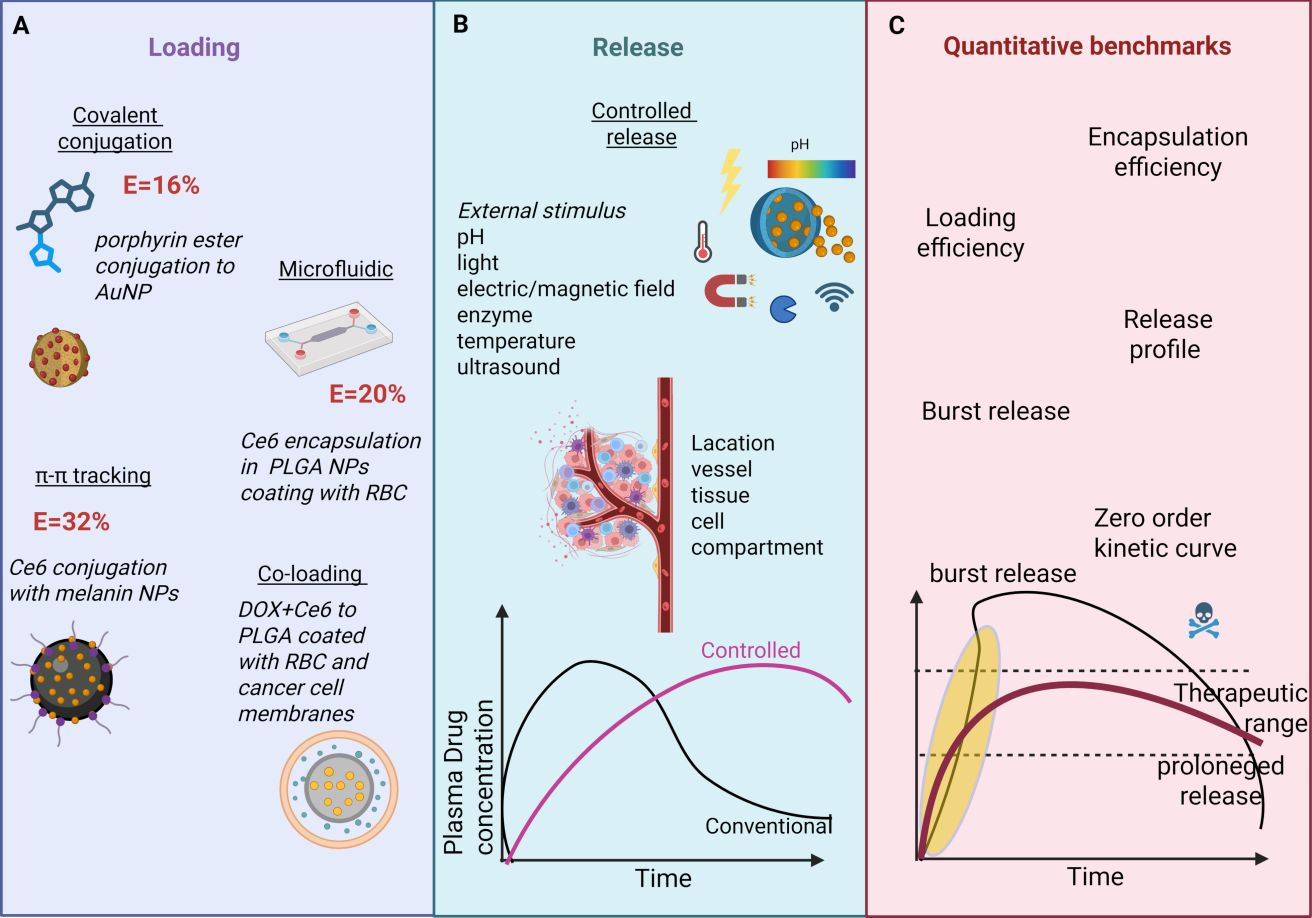
Biomimetic nanoparticle strategies for PS loading and controlled release in PDT. (A) Loading methods; (B) controlled-release triggers; (C) quantitative benchmarks for loading and release estimation. The figure was created in BioRender. Syrocheva, A. (2026) https://biorender.com/cknfimy. This content is not subject to CC BY 4.0.

Beyond efficient loading, controlled PS release from BNPs represents a complementary critical factor governing PDT efficacy within the TME. Biomimetic coatings not only facilitate high payload delivery but also enable stimuli-responsive release mechanisms tailored to TME conditions, including light-triggered bursts (e.g., >70% PS liberation within 5 min via azobenzene linker cleavage under 660 nm irradiation [159]), enzyme-sensitive untethering (MMP-2 hydrolysis of peptide linkers overexpressed in tumor ECM [160]), and biomimetic pH gradients (sustained zero-order release over 24–72 h during endosomal acidification [161]). These strategies ensure spatiotemporal ROS generation while minimizing off-target phototoxicity, as exemplified by membrane-cloaked systems exhibiting reduced initial burst (<20% at 4 h) and prolonged therapeutic windows. These integrated loading/release optimizations are illustrated in Figure 5.

In summary, biomimetic platforms coated with cell membranes or molecules offer a highly promising strategy for the targeted delivery in cancer theranostics, particularly in PDT. By leveraging homotypic recognition and binding to tumor cells, these coatings enhance selective accumulation within tumors while minimizing adverse effects on healthy tissues. Moreover, the integration of diverse therapeutic agents within these BNPs can yield synergistic antitumor effects, thereby improving the overall efficacy of PDT. This approach represents a significant advancement in the development of precise and effective cancer treatments. The precise loading and controlled release of PS into BNPs represent equally pivotal determinants of PDT efficacy, warranting comprehensive analysis alongside encapsulation strategies. While high loading capacities ensure adequate PS payloads for sufficient ROS generation at tumor sites, release kinetics govern bioavailability within the tumor microenvironment, spatiotemporal control of photoactivation, and minimization of off-target phototoxicity during systemic circulation.

### Addressing tumor hypoxia: BNP innovations

The intrinsic hypoxic nature of solid tumors poses a major challenge to PDT efficacy, as limited oxygen availability restricts ROS production. Furthermore, the oxygen consumption during PDT can exacerbate tumor hypoxia, which not only diminishes therapeutic outcomes but also promotes tumor progression, metastasis, and resistance to treatment. Consequently, overcoming tumor hypoxia has become a critical focus in enhancing PDT effectiveness, with numerous strategies being developed to improve oxygen supply, reduce oxygen consumption, or employ oxygen-independent mechanisms to circumvent this limitation. Addressing hypoxia is essential for advancing PDT toward more reliable and potent cancer therapies.

**Figure 6:**
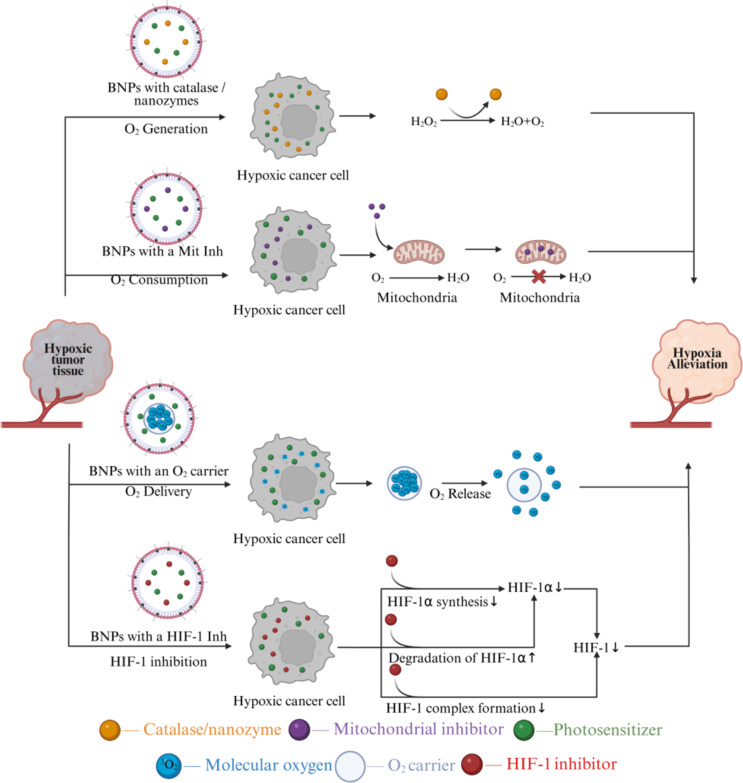
Strategies for overcoming tumor hypoxia limitations in photodynamic therapy through biomimetic nanoparticles engineered as advanced multifunctional nanocarriers. The figure was created in BioRender. Syrocheva, A. (2026) https://biorender.com/4dxq3x2. This content is not subject to CC BY 4.0.

The primary strategies for alleviating tumor hypoxia can be categorized into four main approaches: (i) direct oxygen delivery to the tumor [162–165], (ii) in situ oxygen generation [166–169] within the hypoxic microenvironment, (iii) reduction of oxygen consumption by tumor cells [170–172], and (iv) suppression of hypoxia-inducible factor 1 (HIF-1) activity in the tumor [173–174]. Typically, these nanoparticles consist of a functional core, perfluorocarbons (PFCs) [175–177] or MOFs [165,178–179], and a stabilizing matrix often composed of polymers, lipids, or proteins like human serum albumin (HSA) [177]. Figure 6 schematically illustrates examples of these key strategies for mitigating hypoxia in tumor tissues using BNPs. Table 2 presents the main strategies for using BNPs to mitigate hypoxia in tumors.

**Table 2:** Various strategies for tumor hypoxia alleviation.

System	PS	Source of biomimetic shell	Strategy	Ref

CCm–HSA–ICG–PFTBA	ICG	CCM of 4T1	oxygen delivery via high solubility in perfluorotributylamine (PFTBA), enabling passive release within the tumor to alleviate hypoxia and enhance photodynamic therapy efficacy	[177]
O_2_@UiO-66@ICG@RBC	ICG	RBC	Under 808 nm NIR laser irradiation, the photosensitizer ICG coordinated with UiO-66 generates singlet oxygen that disrupts erythrocyte membranes, while the photothermal effect of ICG induces localized heating to facilitate rapid oxygen release from UiO-66 pores.	[178]
N/P@MCC	PpIX	CCM of 4T1	Catalase immobilized in hollow mesoporous nanospheres decomposes endogenous H_2_O_2_ within the tumor to generate O_2_.	[180]
PM-W_18_O_49_-Met	W_18_O_49_	PM (platelet membrane)	Metformin reduces tumor cell oxygen consumption by inhibiting complex I of the mitochondrial respiratory chain.	[181]
RBCm@Ato-IR780-PFC	IR780	RBC	A perfluorocarbon core within liposomes dissolves high levels of O_2_, releasing it into the hypoxic TME; concurrently, atovaquone inhibits complex III of the mitochondrial respiratory chain, reducing cellular oxygen consumption.	[182]
ARISP	ICG	RBC	Delivery of salidroside, which inhibits HIF-1α, reduces hypoxia in the cell.	[174]

#### Oxygen delivery

To overcome tumor hypoxia and enhance the efficacy of PDT in triple-negative breast cancer, a multifunctional BNP system was developed. The photosensitizer ICG was encapsulated within a HSA matrix to prevent aggregation and supplemented with perfluorotributylamine (PFTBA) as an oxygen carrier. The nanoparticles were further coated with a 4T1 CCM to enable targeting. Direct oxygen delivery to the tumor via PFTBA resulted in a significant reduction of hypoxia, and immunofluorescence staining revealed a tenfold decrease in hypoxic regions within the tumors. In 4T1 cells exposed to NIR laser irradiation, membrane-coated nanoparticles generated singlet oxygen with threefold greater efficiency compared to non-coated nanoparticles. In BALB/c mouse models bearing 4T1 tumors, the membrane-coated nanoparticles preferentially accumulated in tumor tissue, whereas non-coated particles also distributed to the liver and kidneys. Fourteen days post-treatment, a threefold reduction in tumor volume was observed [177].

An alternative strategy has also been described, wherein the photosensitizer ICG is encapsulated within a zirconium-based MOF that functions both as an oxygen reservoir and carrier. The particles were coated with an erythrocyte membrane, which confers immune evasion and enables passive tumor targeting via the EPR effect. Oxygen delivery was mediated directly by the MOF. Studies using murine macrophages (RAW264.7) demonstrated that membrane-coated nanoparticles effectively evade phagocytosis. In three-dimensional MCF-7 cell spheroids, oxygen-saturated nanoparticles increased system cytotoxicity fourfold. In animal models (Balb/c-nu mice bearing MCF-7 tumors), combination treatment resulted in a significant reduction of tumor hypoxia, as demonstrated by photoacoustic imaging showing increased oxygenated hemoglobin levels; also, immunofluorescence staining revealed decreased HIF-1α expression compared to the control group. By day 27 after treatment initiation, the relative tumor volume was 100 times smaller compared to the control group [178].

Another system consisted of ICG and PFTBA loaded into a HSA core, which was subsequently coated with an erythrocyte membrane containing the CD47 protein to enable prolonged circulation (up to 24 h) and immune evasion. In vitro studies using HeLa cells and RAW264.7 macrophages demonstrated that the biomimetic membrane coating reduced macrophage-mediated clearance of the nanoparticles to a half, while maintaining efficient uptake by HeLa cells and preserving phototoxicity upon NIR irradiation. In vivo experiments in a CT-26 tumor mouse model showed that membrane-coated nanoparticles accumulated in tumors at twice the level of their non-coated counterparts. Fourteen days after treatment, tumor size was reduced to a half with PDT alone and 22-fold with combined PDT and PTT [183].

#### In situ oxygen generation

To therapeutically target hypoxic tumors, nanoparticles comprising the PS protoporphyrin IX conjugated with nitrogen-doped graphene quantum dots, catalase for the decomposition of H_2_O_2_ to generate molecular oxygen, and a biomimetic coating derived from 4T1 cell membranes were developed. Oxygen generation occurred directly within the TME. Under hypoxic conditions, BNPs exhibited high ROS production, reduced macrophage uptake, and a twofold decrease in 4T1 cell viability as a result of PDT and PTT. In BALB/c mice bearing 4T1 tumors, nanoparticle accumulation in tumors was fivefold higher compared to the liver, and tumor growth was reduced 15-fold by day 21 relative to the control group. The BNPs demonstrated biodegradability with clearance occurring within 48 h [180].

An effective strategy for suppressing hypoxia in triple-negative breast cancer and lung metastases was achieved using nanoparticles composed of Ce6 combined with catalase for oxygen generation, monophosphoryl lipid A for TLR4 activation, and a 4T1 cell membrane coating to provide homotypic targeting and immune evasion, resulting in circulation times of up to 12 h. The toxicity of the system on 4T1 cells was increased sevenfold compared to free Ce6. In murine tumor models, membrane-coated nanoparticles exhibited a twofold increase in tumor accumulation and reduced tumor hypoxia from 60% to 11%. By day 24 post-treatment, tumor volume was decreased 12-fold. Median survival was extended to 40 days, and the average number of lung metastases was reduced from eleven to two [184].

Currently, nanoparticle platforms for sonodynamic therapy, which utilizes ultrasound rather than light as the external stimulus, are under development for tumor treatment. Systems based on Ag_2_S quantum dots embedded in a Pluronic F-127 matrix, combined with catalase and coated with erythrocyte membranes, have been fabricated. These nanoparticles enabled effective NIR-II imaging. In vitro studies on C26 cells demonstrated that membrane-coated nanoparticles induced 60% cytotoxicity while maintaining active ROS generation under hypoxic conditions. In vivo experiments using C26 tumor-bearing mice showed a twofold increase in preferential tumor accumulation of the coated nanoparticles, with tumor volume reduced threefold by day 21 post-treatment. When combined with phenethyl isothiocyanate, complete tumor eradication was achieved, and survival reached 60% at 40 days [185].

As another example distinct from PDT, chemotherapy under hypoxic conditions was achieved using BNPs: A PLGA-based platform was developed encapsulating DOX and catalase; it was coated with membranes derived from activated M1 macrophages expressing integrins α4 and β1, which mediate targeting via VCAM-1. After 48 h of treatment, biomimetic systems reduced the migration rate of 4T1 cells threefold compared to the control and 1.5-fold compared to free DOX. Furthermore, 4 h after exposure, membrane-coated nanoparticles exhibited a tenfold increase in cellular uptake relative to the control and 1.5-fold higher accumulation compared to free DOX. In vivo, the system resulted in a fivefold reduction in tumor volume by day 21 and a twofold decrease in the number of lung metastases [186].

#### Reduction of oxygen consumption

Nanoparticles based on W_18_O_49_ loaded with metformin were designed to synergistically combine PDT and PTT. These particles were coated with platelet membranes expressing P-selectin for active targeting via PCLP1. Metformin inhibited mitochondrial complex I, thereby decreasing oxygen consumption. In vitro studies on Raji cells demonstrated 80% cytotoxicity. In BALB/c-nude mice bearing Raji lymphoma, the proportion of tumor tissue overexpressing HIF-1α decreased to 7% (compared to 100% in controls), and tumor volume was reduced 5.5-fold by day 12 post-treatment [181].

As an example of a platform addressing hypoxia via dual mechanisms, nanoparticles containing the PS IR780, PFTBA for oxygen delivery, and atovaquone for mitochondrial complex III inhibition were developed and coated with erythrocyte membranes and modified for mitochondrial targeting. This dual approach combined oxygen delivery with reduced oxygen consumption. Under in vitro conditions, these nanoparticles nearly completely inhibited HIF-1α expression and increased ROS generation 1.5-fold compared to free PS. In AGS tumor-bearing mice, tumor volume decreased threefold by day 16, with HIF-1α levels reduced to baseline and pronounced signs of cell death observed [182].

#### Supression of hypoxia-inducible factor 1

In the context of PDT, HIF-1 plays a dual and critical role. The hypoxic state stabilizes and activates HIF-1, a master regulator of cellular responses to low oxygen levels [187]. HIF-1 induces the expression of genes involved in angiogenesis, metabolism, and survival pathways, promoting tumor adaptation and resistance to PDT-induced stress [188]. Notably, PDT can also directly activate HIF-1 signaling, even under normoxic conditions, through prostaglandin pathways and oxidative stress. While HIF-1-driven responses may initially alleviate tumor hypoxia by stimulating angiogenesis, its activation ultimately contributes to therapeutic resistance by enhancing tumor cell survival, metabolic reprogramming, and immunosuppression. Therefore, targeting HIF-1 or its downstream effectors alongside PDT is a strategic approach to mitigate hypoxia-associated limitations.

To suppress HIF-1α and reduce chemoresistance, siRNA targeting HIF-1α was loaded into magnetic nanoparticles coated with a biomimetic membrane derived from macrophages (J774A.1) and cancer cells (4T1). This biomimetic coating enhanced tumor accumulation of the nanoparticles and resulted in a fourfold reduction in HIF-1α expression [173]. ARISP are BNPs developed for targeted PDT under tumor hypoxia conditions. They comprise a core of PLGA loaded with the photosensitizer ICG and salidroside, an inhibitor of HIF-1α synthesis, surrounded by a shell of erythrocyte membranes modified with anti-LDLR antibodies to enable delivery specifically to hypoxic tumor regions. In vitro experiments using the 4T1 cell line under hypoxic conditions demonstrated that ARISP generated ROS at levels 2.6-fold higher than nanoparticles lacking the biomimetic coating and fourfold higher than free ICG, resulting in over a 40-fold increase in cytotoxicity. In vivo studies in 4T1 and MDA-MB-231 mouse models showed that these nanoparticles significantly shortened tumor elimination time. Furthermore, ARISP exhibited prolonged blood circulation for up to 72 h and effectively inhibited both tumor growth and metastasis [174].

#### ROS generation enhancing

Enhancing ROS production upon light activation of PSs is crucial for maximizing the therapeutic efficacy of PDT. Conventional PSs often face intrinsic limitations, including inefficient ROS generation, aggregation, photobleaching, and susceptibility to the hypoxic TME, which severely restrict their clinical performance. BNPs offer a highly promising and versatile platform to overcome these challenges. BNPs not only improve PS delivery but also enable innovative strategies to actively enhance ROS production.

Classical PSs often follow a type-II photosensitization pathway, transferring energy to molecular oxygen to generate singlet oxygen, which is limited in hypoxic tumors. Biomimetic assemblies facilitate a switch to or enhancement of the type-I pathway, which proceeds via electron transfer (superoxide O_2_^−^, hydroxyl radicals) that can form even under low-oxygen conditions. Recent studies demonstrated that biomimetic phosphate-templated supramolecular assemblies of PS promote efficient nanoscale aggregation that blocks energy transfer-type quenching and instead promotes photoinduced charge separation [189]. This favors electron transfer reactions producing superoxide radicals at high efficiency. By converting type-II PSs into type-I supramolecular PSs, biomimetic nanocarriers enhance overall ROS yield, enable ROS generation in hypoxia, and significantly boost PDT efficacy.

The association between PSs and the nanocarrier’s biomimetic components can modulate photophysical and photochemical behavior, thereby controlling ROS generation pathways and efficacy, which depends on the relative probabilities of various excited-state relaxation pathways. The probability of singlet oxygen generation by type-II PSs can be increased by enhancing the transition probability of the PS to the triplet state, with biomimetic nanocarriers influencing this transition and thereby modulating energy transfer to surrounding molecular oxygen [190]. Immobilization or constrained orientation of the PSs within BNPs restrict vibrational and rotational relaxation and shield the PS from quenching by external molecules (e.g., water or biomolecules). Photobleaching and aggregation can also introduce new relaxation pathways that decrease the efficiency of ROS generation. For example, cell membrane coating creates a natural lipid environment that maintains monomeric state and photosensitivity of the PS [112]. Incorporation of plasmonic materials such as gold or silver nanoparticles into biomimetic nanocarriers can enhance local electromagnetic fields near the PS through localized surface plasmon resonance, amplifying light absorption and promoting singlet oxygen generation. Hybrid systems embedding plasmonic silver nanoparticles coated with mesoporous silica and loaded with a PS (e.g., hematoporphyrin IX) exhibit resonance coupling that dramatically increases singlet oxygen production under broad spectral excitation, including NIR wavelengths [191].

### Immune modulation: BNPs empowering PDT-induced tumor immunity

#### PDT-induced immune activation

The immunological potential of PDT was largely unlocked with the discovery of its ability to induce ICD, a regulated form of cell death that activates the adaptive immune system rather than merely eliminating tumor cells [192]. This represents a paradigm shift from early research in the 1990s focused on tumor ablation. The efficacy of immune activation hinges on the presence of tumor-associated antigens (TAAs) and tumor neoantigens (TNAs), which confer antigenicity to cancer cells. However, these antigens alone are typically insufficient to drive robust antitumor immunity without additional signals that recruit and activate antigen-presenting cells. ICD provides precisely this adjuvant-like function through the release of damage-associated molecular patterns (DAMPs); these are molecules normally sequestered within healthy cells and integral to their homeostasis, which, upon extracellular release, act as potent danger signals [193–195]. Key DAMPs include surface-exposed calreticulin (CRT), which promotes phagocytosis [196–197], extracellular ATP, a chemoattractant for motile phagocytes [198–199], released HMGB1, which binds Toll-like receptors (TLRs) to enhance immune activation [200], and heat shock proteins (HSP70/90), which facilitate antigen presentation [201–203]. Collectively, these signals promote tumor antigen uptake, DC maturation, and the cross-priming of cytotoxic T lymphocytes, initiating a systemic antitumor response [204].

The efficacy of PDT in inducing robust ICD is not universal; it critically depends on two main factors, namely, the intracellular localization of the PS and the magnitude and nature of ROS generation [205]. Importantly, ROS should not be regarded as a homogeneous entity; different species, such as singlet oxygen (^1^O_2_), superoxide anions (O_2_^−^•), hydroxyl radicals (•OH), and hydrogen peroxide (H_2_O_2_), exhibit distinct reactivities, diffusion capacities, and half-lives, which collectively determine the spatial extent and biochemical consequences of oxidative damage. The majority of clinically used PSs operate primarily via type-II photochemical reactions, generating singlet oxygen as the dominant reactive species [206]. ^1^O_2_ is highly reactive but extremely short-lived (<0.04 µs in cells [207]), confining its effects to the immediate vicinity of the PS. In contrast, O_2_^−^• and •OH can initiate chain reactions and propagate oxidative damage more broadly [208], while H_2_O_2_, though less reactive, is relatively long-lived and can diffuse across membranes to modulate redox-sensitive signaling pathways, often in an iron- or copper-dependent manner [209–210]. This chemical heterogeneity implies that the subcellular site of ROS generation is as critical as the ROS itself since oxidative stress in one compartment (e.g., endoplasmic reticulum (ER)) can trigger immunogenic signaling, whereas the same species in another (e.g., cytosol) may lead to non-immunogenic collapse or even immunosuppression. Notably, the intensity of oxidative stress directly scales with ICD efficacy; antioxidant interventions consistently reduce DAMP emission and impair adaptive immunity, confirming ROS as non-redundant mediators of immunogenicity.

**Figure 7:**
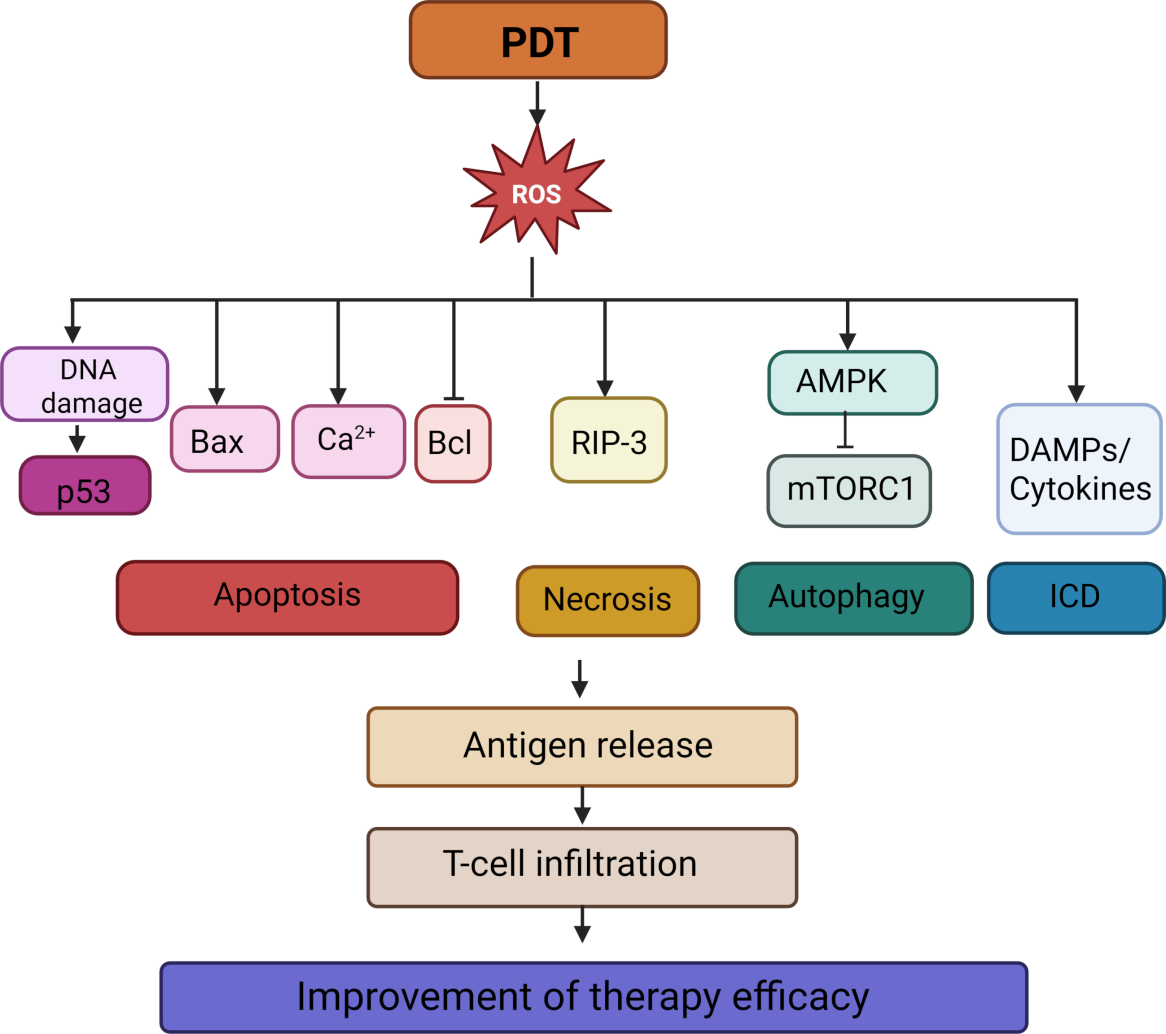
The schematic diagram illustrates the mechanisms by which PDT induces oxidative stress and subsequent cell death, leading to T-cell activation and the initiation of an immune response. The figure was created in BioRender. Syrocheva, A. (2026) https://biorender.com/b9xpatq. This content is not subject to CC BY 4.0.

The selection of the PS and its subcellular target dictates the cell death pathway and the ensuing immunogenic profile through distinct stress-signaling cascades (Figure 7) [211]. For instance, endoplasmic reticulum-targeting PSs (e.g., hypericin) trigger intense ER stress, leading to the activation of the unfolded protein response and the PERK–eIF2α pathway. This cascade promotes the pre-apoptotic translocation of CRT to the plasma membrane, an essential “eat me” signal for dendritic cell recognition, making ER-localized PSs particularly potent inducers of ICD [212–213]. Lysosomal PSs (e.g., Photosens) cause lysosomal membrane permeabilization upon irradiation, releasing cathepsins into the cytosol. This proteolytic burst can activate both caspase-dependent apoptosis and, under conditions of iron accumulation and lipid peroxidation, contribute to ferroptotic cell death, though the resulting DAMP profile is often less coordinated than that induced by ER stress [214–216]. Mitochondrial PSs (e.g., Photofrin) primarily induce rapid mitochondrial outer membrane permeabilization, cytochrome-c release, and swift caspase activation [217]. While this efficiently kills tumor cells, the speed and caspase dominance of this apoptotic pathway often suppress key ICD hallmarks, such as CRT exposure and ATP secretion, resulting in limited dendritic cell activation and weaker adaptive immunity. While caspase-dominated or rapid apoptotic death often fails to elicit robust immunity, lytic forms such as necroptosis typically provoke stronger DAMP release and a more pronounced pro-inflammatory response [218–219]. Additionally, the temporal kinetics of DAMP release alongside the immunological milieu of TME critically influence both the magnitude and quality of the immune response. Sublethal PDT doses can induce a pre-immunogenic cell death phenotype characterized by partial DAMP exposure and modulated cytokine secretion, potentially resulting in either immune enhancement or suppression contingent upon the TME composition.

#### The effect of PDT on immune cells

PDT exerts both direct and context-dependent effects on immune cells, with macrophages playing a central role in modulating of TME and inflammatory responses. Macrophages display polarized phenotypes, including pro-inflammatory, antitumor (M1) and anti-inflammatory, often pro-tumor (M2) states, with tumor-supportive macrophages predominantly exhibiting M2 polarization. PDT-induced ROS can reprogram macrophage polarization from M2 to M1, thereby enhancing antitumor immunity [220]. For example, low-dose PDT using temoporfin-loaded nanoparticles drives repolarization of M2-polarized THP-1 macrophages toward an M1-like phenotype, evidenced by upregulated expression of iNOS, CD86, and TNF-α [221]. Similarly, 5-aminolevulinic acid (5-ALA)-based PDT exhibits selective cytotoxicity toward M2 macrophages, attributable to their higher intracellular accumulation of protoporphyrin IX, while sparing or promoting differentiation of monocyte precursors toward an M1-like state [222]. In vivo, PDT-mediated depletion of M2 tumor-associated macrophages (TAMs) and subsequent infiltration of M1 macrophages have been demonstrated in murine breast cancer models, correlating with improved tumor control [223]. Mechanistically, ROS activate signaling cascades such as COX-2/TREM-1 under inflammatory conditions and ERK/MAPK–NLRP3 inflammasome pathways, which may operate similarly in oncological settings to sustain pro-inflammatory reprogramming [224].

Beyond macrophages, PDT recruits and activates neutrophils, which contribute to early tumor destruction via ROS and NETs [225], and enhances NK cell cytotoxicity through IL-12 and type-I interferons released by activated myeloid cells [226]. DCs are primarily activated indirectly, that is, DAMPs (CRT, ATP, and HMGB1) from PDT-killed tumor cells drive DC maturation and cross-priming of T cells [227–229]. Although high-dose PDT can impair DCs upon direct irradiation, therapeutic regimens predominantly elicit DC activation.

#### BNP-enhanced PDT-immunotherapy synergy

The antitumor immune response in PDT can be potentiated through two primary, non-mutually exclusive mechanisms, that is, first, by intensifying the direct photodynamic effect on cancer cells to robustly induce ICD, and second, by engineering nanocarriers to actively engage and modulate immune cells within the TME via inherent cellular tropism [230]. Nanoparticles cloaked in immune cell membranes, such as those derived from TAMs, exploit the natural homing ability of these cells to the tumor site. For instance, TAM membrane-coated upconversion nanoparticles loaded with Ce6 (NPR@TAMM) have been shown to not only elicit ICD but also to reprogram immunosuppressive M2-like TAMs toward an M1-like, pro-inflammatory phenotype, thereby enhancing CD8^+^ T-cell activation in breast cancer models [231–232]. Another study confirmed that TAM-mimetic nanoparticles significantly increase intratumoral infiltration of CD8^+^ T cells while concomitantly reducing regulatory T-cell populations [233]. An alternative strategy leverages CCM-coated nanoparticles, which achieve homotypic tumor targeting through the preservation of native surface antigens. Yang et al. demonstrated that Ce6-loaded silica nanoparticles coated with gastric CCMs enable precise tumor accumulation, leading to effective PDT and potent ICD induction [125].

Dehaini et al. pioneered the use of erythrocyte-platelet hybrid membranes to confer both prolonged systemic circulation (via CD47) and vascular targeting capabilities (from platelet membranes) [234]. When loaded with the PS verteporfin, these constructs demonstrated superior PDT efficacy in lung cancer models [235]. Collectively, these approaches, spanning immune cells, cancer cells, hybrids, and microbe-inspired biomimetic coatings, represent a versatile toolkit for immuno-photodynamic therapy, as summarized in Table 3.

**Table 3:** BNPs in PDT-induced immune activation.

Membrane source	Immune mechanisms	System	PS	Ref

cancer cell membrane (autologous)	endogenous tumor-associated antigens (TAAs) displayed on membrane serve as in situ vaccine; PDT-induced ICD releases DAMPs → DC uptake of TAAs, maturation, and cross-presentation → potent CD8^+^ T-cell responses and immunological memory	HLP@SiTGF-β1	IR-780	[236]
		Cur/Ce6-MCNPs	curcumin and Ce6	[237]
		CM/SLN/Ce6	Ce6	[238]
		IRCB@M	IR-780	[239]
microbe-inspired platforms	cancer–bacterial hybrids co-display tumor-associated antigens and PAMPs, functioning as self-adjuvanting nanovaccines.	HPDA@OMV-CC	HPDA	[240]
	bacterial membranes deliver PAMPs (e.g., LPS) that activate TLR2/4 on dendritic cells, triggering maturation and pro-inflammatory cytokine release	bacteria-plant hybrid vesicles	thylakoid membranes	[241]
myeloid-derived suppressor cell membrane	homotypic targeting to MDSC-rich TME; PDT/PTT induces ICD while reprogramming immunosuppressive MDSCs → shifts TME toward pro-inflammatory state (↑ TNF-α, IL-12; ↓ TGF-β, IL-10), enhances CD8^+^ T-cell infiltration and M1 macrophage polarization	BP@ Decitabine @MDSCs	black phosphorus	[242]
NK cell membrane	NK cell membrane enables tumor targeting via NKG2D/DNAM-1 and drives pro-inflammatory M1 macrophage polarization; PDT-induced ICD amplifies DAMP release → DC maturation, CD8^+^/CD4^+^ T-cell infiltration, and systemic abscopal immunity	NK-NPs	TCPP	[196]
hybrid membrane (e.g., cancer + dendritic cell)	hybrid cytomembrane from fused cancer and dendritic cells enables homotypic tumor targeting and intrinsic antigen presentation; PDT-driven ICD synergizes with membrane-displayed tumor antigens and co-stimulatory signals → direct DC-like activation, robust CD8^+^ T-cell priming, and durable systemic antitumor immunity (including abscopal effect)	PCN@FM	MOFs	[243]
macrophage membrane (TAM)	TAM membrane coating enables tumor homing and immune compatibility; acts as a CSF1 decoy → blocks CSF1–CSF1R signaling → repolarizes TAMs from M2 to M1 → suppresses IL-10/TGF-β/Arg-1 → enhances antigen presentation, CD8^+^/CD4^+^ T-cell infiltration, IFN-γ production, and abscopal response	NPR@TAMM	UCNP and Rose Bengal	[231]
neutrophil membrane	neutrophil membrane enables ROS-guided homing to inflamed tumors, vascular barrier penetration, and pro-inflammatory immune activation	CR-NML	Ce6	[244]

### Emerging trends and challenges

**Figure 8:**
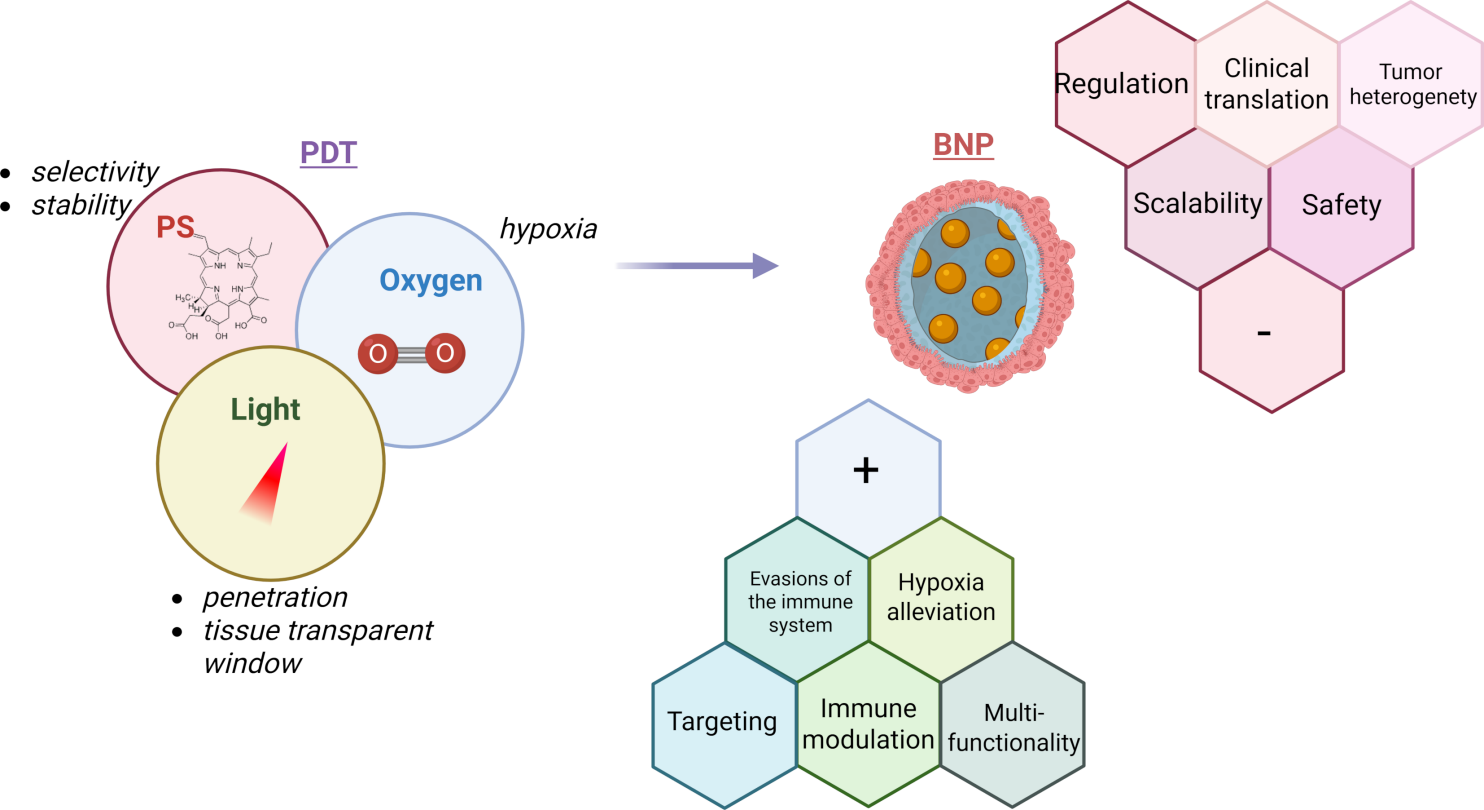
Schematic representation of the advantages and limitations of biomimetic nanoparticles as delivery systems for photosensitizers in photodynamic therapy. Figure 8 was created in BioRender. Syrocheva, A. (2026) https://biorender.com/gero374. This content is not subject to CC BY 4.0.

In recent years, significant progress has been made in the development and application of biomimetic nanoparticle-engineered PDT for cancer treatment. Innovative approaches are advancing the field, including the creation of multifunctional and theranostic nanoparticles, improved methods to counteract tumor hypoxia, and more precise targeting strategies based on tumor characteristics. However, the translation of these technologies into clinical practice faces several challenges, such as production scalability, safety concerns, regulatory requirements, tumor heterogeneity, and cost. This part reviews key emerging trends in biomimetic nanoparticle-mediated PDT and discusses the main obstacles that need to be overcome to enable broader clinical use (Figure 8).

Recent developments in BNP design focus on combining diagnostic and therapeutic functions within a single platform. These theranostic BNPs can carry both imaging agents, such as MRI contrast compounds or photoacoustic dyes, and PSs, allowing for real-time tracking of nanoparticle distribution, tumor oxygen levels, and treatment effectiveness. Additionally, stimuli-responsive systems are being created to release their therapeutic agents in response to specific tumor microenvironment cues like pH changes, redox conditions, or enzyme presence. This improves precise control over drug release, minimizes side effects, and enhances overall efficacy. Moreover, hybrid BNPs are being explored to deliver multiple therapies simultaneously, such as PDT, PTT, chemotherapy, and immunotherapy; this can leverage synergistic effects and address resistance mechanisms.

A notable development in BNP design involves expanding the variety of cell membranes used for nanoparticle coating, such as those derived from platelets, stem cells, and immune cells, alongside RBC and cancer cell membranes. This strategy aims to improve targeting accuracy and tailor BNPs to the specific characteristics of both primary and metastatic tumors. In addition, modifying the nanoparticle surface with tumor-specific ligands, patient-derived antigens, or aptamers helps advance personalized nanomedicine approaches. Alongside these biological advancements, computational methods are increasingly applied to guide BNP design, including predicting physical properties, selecting appropriate surface markers, modeling in vivo distribution, and anticipating treatment responses, which together support more efficient development and clinical translation of BNP-based PDT platforms.

Addressing the challenges posed by the TME is a major focus in current research. To overcome hypoxia, a critical factor limiting PDT effectiveness, BNPs are being developed to include oxygen carriers, such as hemoglobin or perfluorocarbons, or catalytic materials like manganese dioxide and catalase-mimetic nanoparticles that generate oxygen directly within the tumor. At the same time, advances in immune-targeted BNPs enable the combined delivery of immunotherapeutic agents or adjuvants alongside PSs, enhancing antitumor immune responses by promoting ICD and supporting strong activation of T cells.

To overcome the limited tissue penetration of conventional PDT, there is growing interest in NIR-responsive BNPs. Approaches involving upconversion nanoparticles and two-photon excitation are being explored to enhance PS activation at increased depths, thereby expanding the applicability of minimally invasive PDT to a wider variety of tumors.

Clinical translation of BNPs faces challenges including complex large-scale production, ensuring batch consistency, and maintaining surface functionality. Immunogenic risks, especially from non-autologous materials, require thorough in vivo evaluation of biodistribution, clearance, and long-term toxicity. Tumor and patient heterogeneity affect targeting and treatment response, making personalized BNPs promising but difficult to implement broadly. Regulatory guidelines for BNPs are still emerging, and few PDT platforms have reached clinical trials. Additionally, manufacturing costs and the need for advanced imaging and light delivery systems may limit widespread adoption and patient access. Successful resolution of these challenges will be critical for the realization of the full potential of BNP-engineered PDT as a foundational strategy for precision oncology.

While BNPs have shown promising three- to fourfold enhancements in PS delivery and ROS production, often translating to complete tumor regression in preclinical models, the extent to which these improvements represent a qualitative leap over conventional PDT remains an open question. Achieving clinical superiority, particularly for aggressive or deep tumors, may necessitate not merely incremental gains but orders-of-magnitude increases in tumor accumulation (e.g., more than tenfold) and localized ROS generation (e.g., exceeding 10^15^ ROS molecules per cancer cell for apoptosis [245]) to fully compensate for light penetration constraints and hypoxic resistance. For context, conventional Photofrin^®^ PDT achieves 25–86% local control rate at concentrations of 0.78–7.1 μM and fluences of 300–700 J/cm^2^ with ROS yields of 0.70–1.15 mM [246], whereas BNPs often match or exceed this (e.g., fourfold ROS increase, >90% cell killing at 15 μg/mL ICG equivalents) with two- to fivefold reduced doses. Establishing objective quantitative benchmarks, such as standardized metrics for ROS yield per PS dose and comparative survival endpoints against FDA-approved agents, is essential for rigorous evaluation. Emerging preclinical data await phase-I clinical validation to bridge this translational gap.

## Conclusion

BNPs have shown great potential as flexible and effective platforms to improve PDT for cancer treatment. By combining natural cell membrane functions with the adaptable design of nanocarriers, these nanoparticles overcome some limitations of traditional delivery methods. They enhance photosensitizer solubility and stability, reducing common problems like aggregation and poor bioavailability. The biomimetic coating also improves tumor targeting through mechanisms such as homotypic binding and recognition of tumor markers, increasing photosensitizer uptake in cancer cells while protecting healthy tissue and lowering toxicity. Additionally, BNPs can carry oxygen or other features to counteract tumor hypoxia and boost reactive oxygen species production, further improving treatment effectiveness. Altogether, these capabilities work together to increase the selectivity and safety of PDT, enabling more efficient tumor destruction with fewer side effects. However, challenges remain, including the complex and costly process of large-scale production, risks of immune reactions, variability in tumor targeting, limited light penetration into tissue, and regulatory obstacles that need resolution before widespread clinical use. Future work should aim to refine nanoparticle designs for personalized treatment, enhance safety, and combine PDT with immune-based approaches to strengthen anticancer effects. With ongoing research and collaboration, biomimetic nanoparticle-based PDT could become a key tool in personalized cancer therapy, providing safer and more effective treatment options across many tumor types.

## Data Availability

Data sharing is not applicable as no new data was generated or analyzed in this study.
